# Nutritional Ketoacidosis During Incremental Exercise in Healthy Athletes

**DOI:** 10.3389/fphys.2019.00290

**Published:** 2019-03-29

**Authors:** David J. Dearlove, Olivia K. Faull, Edward Rolls, Kieran Clarke, Pete J. Cox

**Affiliations:** ^1^Department of Physiology, Anatomy and Genetics, University of Oxford, Oxford, United Kingdom; ^2^Nuffield Department of Clinical Neurosciences, University of Oxford, Oxford, United Kingdom; ^3^Mathematical Institute, University of Oxford, Oxford, United Kingdom

**Keywords:** ketone, ketoacidosis, exercise, respiratory compensation, ventilatory threshold, lactate accumulation

## Abstract

**Purpose:** Ketosis, achieved through ingestion of ketone esters, may influence endurance exercise capacity by altering substrate metabolism. However, the effects of ketone consumption on acid-base status and subsequent metabolic and respiratory compensations are poorly described.

**Methods:** Twelve athletically trained individuals completed an incremental bicycle ergometer exercise test to exhaustion following the consumption of either a ketone ester [*(R)*-3-hydroxybutyrate-*(R)*-1,3-butanediol] or a taste-matched control drink (bitter flavoured water) in a blinded, cross-over study. Respiratory gases and arterialised blood gas samples were taken at rest and at regular intervals during exercise.

**Results:** Ketone ester consumption increased blood D-β-hydroxybutyrate concentration from 0.2 to 3.7 mM/L (*p* < 0.01), causing significant falls versus control in blood pH to 7.37 and bicarbonate to 18.5 mM/L before exercise. To compensate for ketoacidosis, minute ventilation was modestly increased (*p* < 0.05) with non-linearity in the ventilatory response to exercise (ventilatory threshold) occurring at a 22 W lower workload (*p* < 0.05). Blood pH and bicarbonate concentrations were the same at maximal exercise intensities. There was no difference in exercise performance having consumed the ketone ester or control drink.

**Conclusion:** Athletes compensated for the greater acid load caused by ketone ester ingestion by elevating minute ventilation and earlier hyperventilation during incremental exercise.

## Introduction

Ketosis is our metabolic adaptation to starvation (Cahill, 1970). The production of the lipid-derived ketone bodies, D-β-hydroxybutyrate (D-βHB) and acetoacetate, prolongs survival during starvation by providing a supplementary oxidisable carbon source for nerve tissue, subsequently slowing the catabolism of finite glycogen and gluconeogenic skeletal muscle (Owen et al., 1967; Cahill, 1970). Supplementing athletes with nutritional ketones mimics some of the advantageous aspects of starvation ketosis by reducing glycolysis and increasing fat oxidation in working skeletal muscle (Cox et al., 2016). These ketone-mediated alterations in substrate metabolism may improve human endurance exercise capacity in some contexts (Cox et al., 2016).

β-hydroxybutyrate and acetoacetate are weak organic acids. As with starvation, where serum ketone levels plateau at approximately 7.5 mM/L (Robinson and Williamson, 1980), high circulating concentrations of nutritional ketones causes a mild metabolic acidosis (ketoacidosis) (Stubbs et al., 2017). The acidaemia resulting from prolonged starvation or nutritional ketosis is distinctly different to that observed in uncontrolled endogenous ketoacidosis, such as diabetic crisis, where ketone concentrations of ≥20 mM/L may cause blood pH to fall below 6.9 (Koul, 2009).

Human physiology is adept at accommodating the metabolic acidosis associated with high-intensity exercise (Wasserman et al., 1973) or mild ketosis (Rubini et al., 2017; Stubbs et al., 2017). However, the metabolic and respiratory compensations for a combined exercise induced lactic acidosis and nutritional ketoacidosis are poorly described.

## Materials and Methods

### Participants

Twelve healthy athletes (9 males, 3 females; age 28.0 ± 1.6 year; weight 77.8 ± 3.5 kg; VO_2max_ 4.4 ± 0.2 L/min) undertaking a minimum of 6 hr of training per week in endurance sports (rowing cycling, running or swimming) participated in this study. Ethical approval was granted by the Oxfordshire Clinical Research Ethics Committee. All participants provided written, informed consent.

### Protocol

**FIGURE 1:**
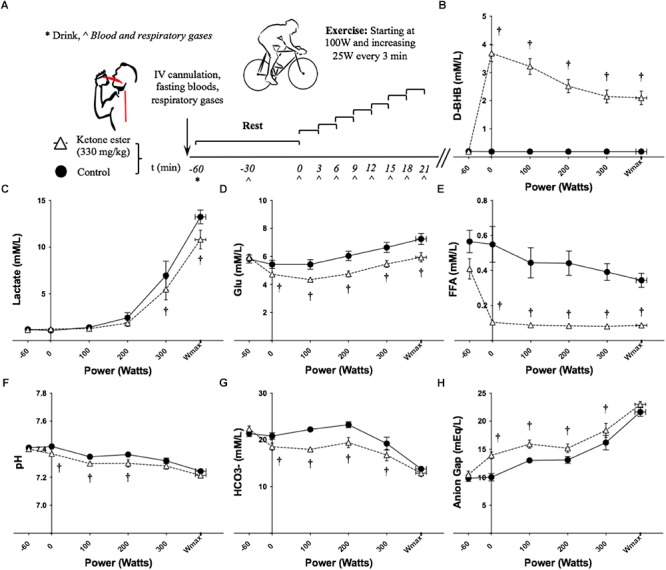
Effects of KE ingestion on blood metabolites and gases at rest and during incremental intensity exercise (*n* = 12). **(A)** Study protocol **(B)** Plasma D-βHB concentration **(C)** Plasma lactate concentration **(D)** Plasma glucose concentration **(E)** Plasma FFA concentration **(F)** Blood pH **(G)** Blood HCO_3_- concentration **(H)** Anion gap. All data are means ± SEM. ^†^Significant difference between ketone ester and control conditions (*p* < 0.05).

Following an overnight fast, subjects performed incremental exercise tests on an electronically braked bicycle ergometer (Ergoline, Germany) on two occasions separated by approximately 1 week (Figure 1A). Exercise began at 100 W, and increased by 25 W every 3 min until exhaustion. This protocol has been shown to reliably and reproducibly identify differences in respiratory gas parameters (Zhang et al., 1991). Prior to commencing exercise, subjects consumed either a ketone ester [KE, 330 mg/kg body weight of *(R)*-3-hydroxybutyl *(R)*-3-hydroxybutyrate] containing drink, or a control beverage (bitter flavoured water). Participants undertook testing in a single-blinded, randomised and counterbalanced, cross-over design.

### Blood Metabolites

Upon arrival, a 22-gauge catheter was inserted retrogradely into a dorsal vein of the hand. Blood samples (1 mL) were drawn for blood gas measurements before and during exercise via the heated hand method (Nauck et al., 1992). Samples were immediately analysed using a benchtop blood gas analyser (Radiometer, Denmark). Calculations of arterial pH and bicarbonate (HCO_3_-) were made using custom MATLAB scripts (MathWorks Inc., United States) (see Supplementary Materials and Methods for equations and Supplementary Table S3 for the uncorrected pH and HCO_3_- values). Concurrent 2 mL blood samples were drawn for analysis of blood metabolites. Samples were immediately stored on ice, centrifuged (3,600 RPM for 10 min), and subsequently stored at -25°C until further analysis. Glucose, non-esterified fatty acids (FFA) and lactate were assayed using a commercial automated bench-top analyser (ABX Pentra, France). Insulin assays were performed using ELISA kits (Mercodia, Sweden). Blood D-βHB was immediately assayed using a portable analyser (Abbott Laboratories Ltd., United Kingdom). Anion gaps were calculated as previously described (Kraut and Madiast, 2007).

### Respiratory Gas Measures and Threshold Determination

Recording of oxygen consumption and minute ventilation (VE) (Cortex Biophysik, Germany) was performed at rest and during exercise. During exercise, a 30 s average of the last min of each interval was used to represent steady state respiratory gas values. The ventilatory threshold (T_vent_) was calculated using the V-slope method (Beaver et al., 1986). A second blinded investigator verified all T_vent_ estimations. In the case of significant discordance (defined as >5% difference in power (W) at the T_vent_), a third blinded investigator acted as arbiter. The maximum power output achieved (W_max_) was calculated as previously described (Cox et al., 2016). Changes in lactate accumulation in response to incremental exercise were determined as the workload (W) at which lactate increased by 1 mM/L above baseline levels, and workload at the blood lactate of 4 mM/L. These were assessed using freely available software (Newell et al., 2007).

### Statistical Analysis

Statistical analysis was performed using GraphPad Prism (GraphPad Software Inc., United States). Following testing to ensure sphericity assumptions were not violated, blood metabolites and gases and respiratory gas data were analysed using a 2-way, repeated measures ANOVA. *Post hoc* comparisons were performed using Bonferroni corrections. Comparisons of W_max_ and workloads corresponding to the T_vent_ and lactate accumulation markers were assessed using Student’s paired *t*-tests. All data are presented as means ± SEM. A *p*-value of <0.05 was taken to indicate statistical significance.

## Results

### Blood Metabolites and Insulin

Overnight fasted D-βHB levels were 0.2 ± 0.0 mM/L in both conditions at baseline. D-βHB increased significantly to 3.7 ± 0.3 mM/L following KE consumption and remained significantly elevated throughout exercise (Figure 1B and Supplementary Table S1). Blood lactate was significantly lower at 300 W and W_max_ having consumed the KE drink (Figure 1C and Supplementary Table S1). Glucose and FFA were the same at baseline, but were significantly lower following KE ingestion before and during exercise (Figure 1D,E and Supplementary Table S1). No differences in blood insulin concentration were found (data not shown).

### Arterialized Blood Gases

Blood pH was the same at baseline (control = 7.41 ± 0.01, KE = 7.40 ± 0.18). Following ingestion of the KE, pre-exercise blood pH fell significantly versus control to 7.37 ± 0.01 (Figure 1F and Supplementary Table S1). Blood pH remained significantly lower during ketosis at 100 W and 200 W, but not at 300 W or W_max_.

Blood HCO_3_- was the same at baseline (control = 21.4 ± 0.63 mM/L, KE = 22.3 ± 0.67 mM/L). The increased H^+^ concentration following KE ingestion caused blood HCO_3_- to fall significantly versus control to 18.5 ± 0.84 mM/L pre-exercise (Figure 1G and Supplementary Table S1). During exercise, blood HCO_3_- remained significantly lower in the KE condition at 100, 200, and 300 W, but not at W_max_.

The anion gap was 10 ± 2 mEq/L at baseline in both conditions. Following ingestion of the KE, the pre-exercise anion gap increased significantly versus control to 14 ± 2 mEq/L and remained significantly higher during exercise at 100, 200, and 300 W, but not at W_max_ (Figure 1H and Supplementary Table S1). The anion gap was the same for both conditions when blood D-βHB concentration was accounted for.

### Cardiorespiratory Measures

**FIGURE 2:**
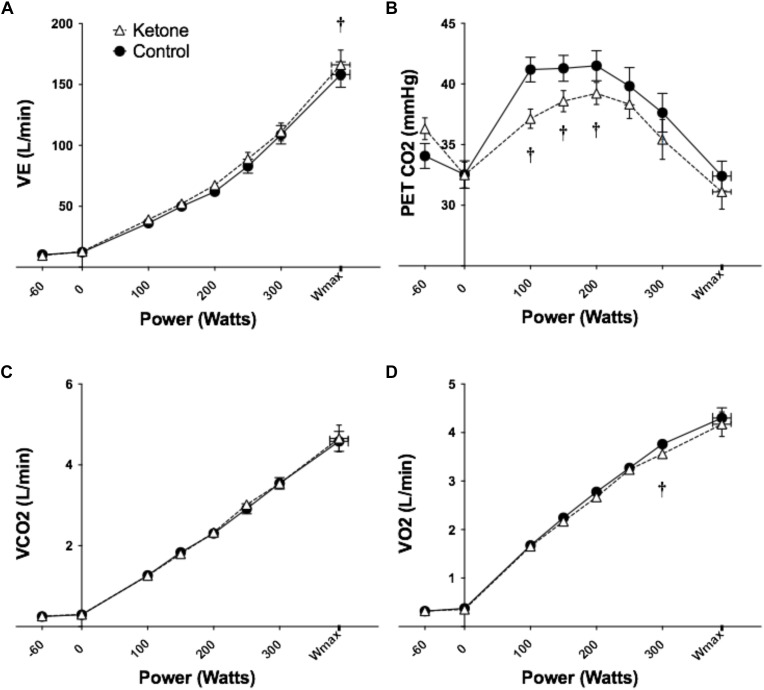
Effects of KE ingestion on blood metabolites and gases at rest and during incremental intensity exercise (*n* = 12). **(A)** V_E_ **(B)** PETCO_2_ **(C)** VCO_2_ **(D)** VO_2_. All data are means ± SEM. ^†^Significant difference between ketone ester and control conditions (*p* < 0.05).

V_E_ was greater in KE versus control only at W_max_ (Figure 2A and Supplementary Table S2). The increased respiratory drive following KE ingestion lowered the partial pressure of end tidal CO_2_ (P_ET_CO_2_) (used as a surrogate for the partial pressure of CO_2_ in the blood) with significant differences at 100, 150, and 200 W (Figure 2B and Supplementary Table S2). Consequently, the partial pressure of end tidal O_2_ (P_ET_O_2_) was greater following consumption of the KE drink (Supplementary Table S2). The volume of carbon dioxide expelled (VCO_2_) was not different between conditions and the volume of oxygen consumed (VO_2_) was lower in the KE condition at 300 W only (Figure 2D and Supplementary Table S2). There were no differences in heart rates (data not shown).

### Physical Performance

Exercise performance (W_max_) was the same in KE (393 ± 22 W) and control (389 ± 20 W). The workload corresponding to T_vent_ was significantly lower in KE (238 ± 13 W) than control (260 ± 15 W). Workload corresponding to a 1 mM/L rise in lactate above baseline was not significantly different between KE (272 ± 17 W) and control (252 ± 22 W). However, workload at the fixed blood lactate level of 4 mM/L was higher in KE (323 ± 65 W) versus control (298 ± 75 W).

## Discussion

Here, we found that nutritional ketoacidosis resulted in compensatory metabolic and respiratory changes to accommodate the acid load, both at rest and during exercise.

V_E_ was elevated following KE consumption at rest and during exercise, with differences being significant at W_max_. As would be expected, this led to reductions in P_ET_CO_2_. The magnitude of these changes in the context of the dramatic (>10 fold) increases in ventilation with exercise are relatively small, representing ∼5% of total ventilation.

Presumably as a result of the additional acid load caused by KE consumption, the onset of exercise hyperventilation [non-linearity in V-slope regression or T_vent_ (Beaver et al., 1986)], occurred at a lower workload in the KE drink. T_vent_ is commonly used to quantify exercise tolerance in athletic and clinical cohorts (Hopker et al., 2011). Our results show this measure is confounded in nutritional ketosis by lower pre-exercise pH, with threshold workloads being significantly underestimated.

KE ingestion lowered blood pH and HCO_3_- and increased the anion gap at rest and during submaximal exercise. The elevated anion gap during ketosis was due to greater D-βHB concentrations, as no differences were observed when these anions were accounted for. However, at higher exercise intensities, where >2–4 mM/L increases in lactate occurred, no differences in pH, HCO_3_- or the anion gap were observed. This suggests that as exponential increases in glycolysis and commensurate lactate production occur during high intensity exercise, the magnitude of effect exogenous keto-acids exert relative to lactate becomes insignificant.

A known metabolic effect of ketosis is to constrain glycolysis, resulting in reduced lactate production during exercise (Cox et al., 2016). This “acid-sparing” action may explain the convergence in acid base profiles with increasing exercise intensity. Such an action has a sound teleological basis in starvation metabolism, where glucose conservation and pH homeostasis are challenged by the need to forage. In this context, the inhibition of glycolysis during exercise-induced acidosis is logical, it being advantageous to inhibit an acid producing pathway.

Sutton et al. (1981) found the rate of glycogen utilisation was lower during acidaemia than alkalaemia or normal pH, suggesting lower blood lactate levels during ketosis could, in part, arise from inhibition of glycolysis by the accompanying acidaemia. Here, significant differences in blood lactate at 300 W and W_max_ coincided with a convergence of blood pH and HCO_3_-. As such, the greatest difference in blood lactate concentrations was simultaneous with the smallest differences in pH and HCO_3_- concentration. Also, the relative rate of blood lactate appearance was lower following KE drink consumption, even during workloads where pH and HCO_3_- were similar, suggesting that the metabolic control of glycolysis (and thus lactate) by ketones remains the dominant action during exercise, rather than pH. Similarly, potentially advantageous alterations in exercise metabolism resulting from ketosis may, in part, explain why work inducing acidaemia via ingestion of ammonium chloride mildly impaired performance, whereas no differences were observed here. However, pre-exercise pH values were lower in these studies (all <7.30, compared to 7.37 in this study) (McCartney et al., 1983; Kowalchuk et al., 1984; George and Mac Laren, 1988; Brien and McKenzie, 1989), which may further account for the relative lack of effect on exercise tolerance here.

Whilst the perturbations to acid-base homeostasis accompanying a mean blood D-βHB concentration of 3.7 mM/L did not impair physical performance, it remains unclear whether this is the same at higher KE concentrations. Therefore, individuals interested in using supplemental ketones for athletic performance should be aware that higher ketone body concentrations are not necessarily better, and may even be deleterious to performance.

Finally, the cohort of athletes studied here have, by nature of their training, developed a large physiological capacity to accommodate perturbations in both acid-base balance and cardiorespiratory stressors. Whether untrained people, or those with chronic medical conditions, are equally capable of compensating for a concurrent ketoacidosis and lactic acidosis during exercise is not known.

## Conclusion

Healthy, athletically trained participants compensated for ketoacidosis during incremental intensity exercise through an increased and earlier respiratory compensation, without a deterioration in exercise performance at these ketone concentrations.

## Author Contributions

DD, OF, and PC were involved in the experimental design, data collection, data analysis, and manuscript preparation. ER assisted with data analysis and manuscript preparation. KC was involved in the manuscript preparation and support for the study. All authors reviewed the manuscript.

## Conflict of Interest Statement

The intellectual property and patents covering the uses of ketone bodies and esters are owned by BTG Ltd., The University of Oxford, the NIH and TdeltaS Ltd. Should royalties ever accrue from these patents, KC and PC as named inventors may receive a share of royalties as determined by the terms of the respective institutions. KC is director of TdeltaS Ltd., a spin out company of the University of Oxford, to develop and commercialise products based on the ketone ester. DD is a current employee of TdeltaS Ltd. and OF a former employee. The remaining author declares that the research was conducted in the absence of any commercial or financial relationships that could be construed as a potential conflict of interest.

## References

[B1] Beaver W. L. Wasserman K. Whipp B. J. (1986). A new method for detecting anaerobic threshold by gas exchange. *J. Appl. Physiol.* 60 2020–2027. 10.1152/jappl.1986.60.6.2020 3087938

[B2] Brien D. M. McKenzie D. C. (1989). The effect of induced alkalosis and acidosis on plasma lactate and work output in elite oarsmen. *Eur. J. Appl. Physiol. Occup. Physiol.* 58 797–802. 10.1007/BF02332209 2548862

[B3] Cahill G. F. (1970). Starvation in man. *N. Engl. J. Med.* 320 668–675. 10.1056/NEJM197003192821209 4915800

[B4] Cox P. J. Kirk T. Ashmore T. Willerton K. Evans R. Smith A. (2016). Nutritional ketosis alters fuel preference and thereby endurance performance in athletes. *Cell Metab.* 24 256–268. 10.1016/j.cmet.2016.07.010 27475046

[B5] George K. P. Mac Laren D. P. M. (1988). The effect of induced alkalosis and acidosis on endurance running at an intensity corresponding to 4 mM blood lactate. *Ergonomics* 31 1639–1645. 10.1080/00140138808966813 3229410

[B6] Hopker J. G. Jobson S. A. Pandit J. J. (2011). Controversies in the physiological basis of the “anaerobic threshold” and their implications for clinical cardiopulmonary exercise testing. *Anaesthesia* 66 111–123. 10.1111/j.1365-2044.2010.06604.x 21254986

[B7] Koul P. B. (2009). Diabetic ketoacidosis: a current appraisal of pathophysiology and management. *Clin. Pediatr.* 48 135–144. 10.1177/0009922808323907 19023105

[B8] Kowalchuk J. Heigenhauser G. Jones N. (1984). Effect of pH on metabolic and cardiorespiratory responses during progressive exercise. *J. Appl. Physiol. Respir. Environ. Exerc. Physiol.* 57 1558–1563. 10.1152/jappl.1984.57.5.1558 6520052

[B9] Kraut J. A. Madiast N. E. (2007). Serum anion gap: its uses and limitations in clinical medicine. *Clin. J. Am. Soc. Nephrol.* 2 162–174. 10.2215/CJN.03020906 17699401

[B10] McCartney N. Heigenhauser G. J. Jones N. L. (1983). Effects of pH on maximal power output and fatigue during short-term dynamic exercise. *J. Appl. Physiol.* 55 225–229. 10.1152/jappl.1983.55.1.225 6885575

[B11] Nauck M. A. Liess H. Siegel E. G. Niedmann P. D. Creutzfeldt W. (1992). Critical evaluation of the “heated-hand-technique” for obtaining “arterialized” venous blood: incomplete arterialization and alterations in glucagon responses. *Clin. Physiol.* 12 537–552. 10.1111/j.1475-097X.1992.tb00357.x 1395446

[B12] Newell J. Higgins D. Madden N. Cruickshank J. Einbeck J. Mcmillan K. (2007). Software for calculating blood lactate endurance markers. *J. Sports Sci.* 2512 1403–1409. 10.1080/02640410601128922 17786693

[B13] Owen O. E. Morgan A. P. Kemp H. G. Sullivan J. M. Herrera M. G. Cahill G. F. (1967). Brain metabolism during fasting. *J. Clin. Invest.* 46 1589–1595. 10.1172/JCI105650 6061736 PMC292907

[B14] Robinson A. Williamson D. (1980). Physiological roles of ketone bodies as substrates and signals in mammalian tissues. *Physiol. Rev.* 60 143–187. 10.1152/physrev.1980.60.1.143 6986618

[B15] Rubini A. Bosco G. Lodi A. Cenci L. Parmagnani A. Grimaldi K. (2017). Effects of twenty days of the ketogenic diet on metabolic and respiratory parameters in healthy subjects. *Lung* 193 939–945. 10.1007/s00408-016-9958-0 26410589

[B16] Stubbs B. J. Cox P. J. Evans R. D. Santer P. Miller J. J. Faull O. K. (2017). On the metabolism of exogenous ketones in humans. *Front. Physiol.* 8:848 10.3389/fphys.2017.00848 PMC5670148 29163194

[B17] Sutton J. Jones N. Toews C. (1981). Effect of pH on muscle glycolysis during exercise. *Clin. Sci.* 61 331–338. 10.1042/cs0610331 7261554

[B18] Wasserman K. Whipp B. J. Koyal S. N. Beaver W. L. (1973). Anaerobic threshold and respiratory gas exchange during exercise. *J. Appl. Physiol.* 35 236–243. 10.1152/jappl.1973.35.2.236 4723033

[B19] Zhang Y. Johnson M. Chow N. Wasserman K. (1991). Effect of exercise testing protocol on parameters of aerobic function. *Med. Sci. Sports Exerc.* 23 625–630. 10.1249/00005768-199105000-00016 2072842

