# Supplementary material for: Nutritional Ketoacidosis During Incremental Exercise in Healthy Athletes
Source: Front Physiol. 2019 Mar 29;10:290. doi: 10.3389/fphys.2019.00290 (PMC6450328; doi:10.3389/fphys.2019.00290)
Supplement: Supplementary file 1 [file Data_Sheet_1.PDF]

Supplementary Material

Nutritional ketoacidosis during incremental exercise in healthy athletes

Authors: David J Dearlove[1*], Olivia K Faull[1,2], Edward Rolls[3], Kieran Clarke[1], Pete J Cox[1]

[1]Department of Physiology, Anatomy & Genetics, University of Oxford, Oxford, UK

[2]Nuffield Department of Clinical Neurosciences, University of Oxford, Oxford, UK

[3]Mathematical Institute, University of Oxford, Oxford, UK

Corresponding author:

David Dearlove

Email: david.dearlove@dpag.ox.ac.uk

Supplementary materials and methods

MATLAB script for arterialised blood-gas corrections

A 'buffer line' was drawn through the venous pH and HCO3- values (denoted as venpH and venHCO3 respectively) with a gradient of *–(8.2 + 9.2 * [Hb].* The buffer line was then shifted to the left by a distance of 0.1 * (0.98 – SvO2), where SvO2 is the oxygen saturation in venous blood. This shift represents the increased saturation of haemoglobin in arterialised blood and, therefore, reduced buffering capacity. This line is given by the equation: $[HCO_3^-]$ =-*(8.2+9.2*Hb)*pH+venHCO3-+a*venpH-a*0.1*(0.98-Svo2).*

The intersection point between the second buffer line and the line given by the Henderson-Hasselbalch equation $pH = pK + \log_{10}([HCO_3^-]/(\alpha.P_{CO2}))$ in the Davenport diagram was sought by rearranging the equation: $[HCO_3^-] = \alpha.P_{CO2}.10^{\wedge}(pH\text{-}pK)$.

The root-finding method fsolve on MATLAB was applied to find the point where these two curves meet, which gives the arterial values for $[HCO_3^-]$ and pH.

Supplementary tables

Table S1: Blood measures at rest and during incremental intensity exercise (n=12) following KE or control drinks. All data are means ± SEM. †significant main effect of intervention (KE versus control), *significant interaction effect (intervention*power). Statistical significance considered $p<0.05$.

	Measurement	Ketone Ester	Control	Δ	t	DF	*p*
**D-βHB**	Baseline	0.2 ± 0.0	0.2 ± 0.0	0.0	0.06	55	>0.05
**(mM/L)**	Pre-Exercise	3.7 ± 0.3	0.2 ± 0.0	3.5	25.63	55	<0.01
†*	100W	3.2 ± 0.3	0.2 ± 0.0	3.0	22.21	55	<0.01
	200W	2.5 ± 0.2	0.2 ± 0.0	2.3	17.07	55	<0.01
	300W	2.1 ± 0.3	0.2 ± 0.0	1.9	14.38	55	<0.01
	Wmax	2.1 ± 0.2	0.2 ± 0.0	1.9	14.01	55	<0.01
**Lactate**	Baseline	1.2 ± 0.1	1.2 ± 0.2	0.0	0.21	55	>0.05
**(mM/L)**	Pre-Exercise	1.2 ± 0.2	1.0 ± 0.2	0.2	0.49	55	>0.05
†*	100W	1.3 ± 0.3	1.4 ± 0.3	-0.1	0.42	55	>0.05
	200W	1.9 ± 0.6	2.4 ± 1.0	-0.5	1.82	55	>0.05
	300W	5.5 + 2.1	7.0 ± 3.2	-1.5	4.67	55	<0.01
	Wmax	10.8 ± 1.2	13.3 ± 0.8	-2.5	7.63	55	<0.01
**Glucose**	Baseline	5.8 ± 0.3	5.6 ± 0.4	0.2	0.42	55	>0.05
**(mM/L)**	Pre-Exercise	4.7 ± 0.1	5.2 ± 0.3	-0.5	2.96	55	<0.05
†*	100W	4.3 ± 0.2	5.3 ± 0.6	-1.0	4.53	55	<0.01
	200W	4.8 ± 0.2	6.0 ± 0.6	-1.2	5.40	55	<0.01
	300W	5.4 ± 0.3	6.5 ± 0.7	-1.1	4.91	55	<0.01
	Wmax	6.0 ± 0.4	7.3 ± 0.5	-1.3	5.47	55	<0.01
**FFA**	Baseline	0.41 ± 0.11	0.57 ± 0.14	-0.16	0.42	55	>0.05
**(mM/L)**	Pre-Exercise	0.11 ± 0.03	0.58 ± 0.22	-0.47	2.96	55	<0.05
†*	100W	0.09 ± 0.02	0.46 ± 0.16	-0.37	4.53	55	<0.01
	200W	0.08 ± 0.02	0.45 ± 0.13	-0.37	5.40	55	<0.01
	300W	0.08 ± 0.01	0.41 ± 0.09	-0.33	4.91	55	<0.01
	Wmax	0.09 ± 0.01	0.35 ± 0.05	-0.26	5.47	55	<0.01
**pH**	Baseline	7.40 ± 0.18	7.41 ± 0.01	-0.01	0.67	55	>0.05
†	Pre-Exercise	7.37 ± 0.01	7.42 ± 0.01	-0.05	3.71	55	<0.01
	100W	7.30 ± 0.01	7.35 ± 0.01	-0.05	3.45	55	<0.01

	200W	7.30 ± 0.03	7.36 ± 0.01	-0.06	4.47	55	<0.01
	300W	7.28 ± 0.03	7.32 ± 0.02	-0.04	2.62	55	>0.05
	Wmax	7.21 ± 0.02	7.24 ± 0.01	-0.03	2.12	55	>0.05
**HCO_3^- (mM/L) †***	Baseline	22.3 ± 0.7	21.4 ± 0.6	0.9	1.35	55	>0.05
	Pre-Exercise	18.5 ± 0.8	20.8 ± 0.7	-2.3	3.25	55	<0.05
	100W	18.0 ± 0.5	22.2 ± 0.4	-4.2	6.01	55	<0.01
	200W	19.4 ± 1.1	23.2 ± 0.6	-3.8	5.40	55	<0.01
	300W	16.8 ± 1.3	19.7 ± 1.4	-2.9	3.47	55	<0.01
	Wmax	12.9 ± 0.8	13.8 ± 0.4	-0.9	1.13	55	>0.05
**Anion gap (mEq/L) †***	Baseline	10.5	9.8	0.7	1.00	50	>0.05
	Pre-Exercise	13.9	10.0	3.9	6.13	50	<0.01
	100W	15.9	13.0	2.9	4.56	50	<0.01
	200W	15.2	13.1	2.1	3.28	50	<0.01
	300W	18.4	16.2	2.2	3.42	50	<0.01
	Wmax	23.0	21.6	1.4	2.14	50	>0.05

Table S2: Cardiorespiratory measures in control and KE conditions at rest and during incremental intensity exercise (n=12) following KE or control drinks. All data are means ± SEM. †significant main effect of intervention (KE versus control), *significant interaction effect (intervention*power). Statistical significance considered *p*<0.05.

	Measurement	Ketone Ester	Control	Δ	t	DF	*p*
**VE (L/min)** †	Baseline	9.6 ± 0.7	10.3 ± 0.9	-0.7	0.28	127	>0.05
	Pre-Exercise	12.9 ± 1.0	12.3 ± 1.2	0.6	0.20	127	>0.05
	100W	39.2 ± 1.9	36.2 ± 2.1	3.0	1.11	127	>0.05
	150W	52.1 ± 2.0	49.9 ± 2.4	2.2	0.82	127	>0.05
	200W	67.4 ± 3.1	62.0 ± 2.7	5.4	2.04	127	>0.05
	250W	88.9 ± 5.3	82.9 ± 5.7	6.0	2.25	127	>0.05
	300W	111.1 ± 7.9	108.7 ± 7.7	2.4	0.88	127	>0.05
	Wmax	166.1 ± 12.1	158.2 ± 10.4	7.9	2.98	127	<0.05
**$P_{ET}CO_2$ (mmHg)** †*	Baseline	36.3 ± 0.90	34.1 ± 1.03	2.2	2.795	77	>0.05
	Pre-Exercise	32.5 ± 0.45	32.5 ± 1.13	0.0	0.05	77	>0.05
	100W	37.1 ± 0.794	41.2 ± 1.02	-4.1	5.04	77	<0.01
	150W	38.6 ± 0.88	41.3 ± 1.08	-2.7	3.37	77	<0.01
	200W	39.2 ± 0.912	41.5 ± 1.24	-2.3	2.84	77	<0.05
	250W	38.3 ± 1.13	39.8 ± 1.54	-1.5	1.86	77	>0.05
	300W	36.0 ± 1.69	37.7 ± 1.74	-1.7	2.74	77	>0.05
	Wmax	31.1 ± 1.43	32.4 ± 1.24	-1.3	1.62	77	>0.05
**$P_{ET}O_2$ (mmHg)** †	Baseline	108.4 ± 0.3	108.5 ± 2.6	-0.1	0.04	77	>0.05
	Pre-Exercise	108.9 ± 6.4	110.1 ± 2.1	-1.2	0.46	77	>0.05
	100W	104.7 ± 1.5	99.5 ± 1.9	5.2	1.96	77	>0.05
	150W	107.0 ± 1.36	102.0 ± 1.63	5.0	1.89	77	>0.05
	200W	108.3 ± 1.4	102.3 ± 1.7	6.0	2.25	77	>0.05
	250W	111.5 ± 1.73	106.7 ± 2.11	4.8	1.83	77	>0.05
	300W	115.7 ± 2.1	110.6 ± 2.4	5.1	1.91	77	>0.05
	Wmax	124.2 ± 1.3	120.6 ± 1.2	3.6	1.37	77	>0.05
**VCO_2 (L/min)**	Baseline	0.3 ± 0.0	0.2 ± 0.0	0.1	0.12	77	>0.05
	Pre-Exercise	0.3 ± 0.0	0.3 ± 0.0	0.0	0.08	77	>0.05
	100W	1.3 ± 0.1	1.3 ± 0.1	0.0	0.08	77	>0.05
	150W	1.8 ± 0.1	1.8 ± 0.1	0.0	0.44	77	>0.05
	200W	2.3 ± 0.1	2.3 ± 0.1	0.0	0.32	77	>0.05
	250W	3.0 ± 0.1	3.0 ± 0.1	0.0	1.25	77	>0.05
	300W	3.5 ± 0.1	3.5 ± 0.1	0.0	0.08	77	>0.05

	Wmax	4.7 ± 0.3	4.6 ± 0.2	0.1	0.87	77	>0.05
**VO_2 (L/min)**	Baseline	0.3 ± 0.0	0.3 ± 0.1	0.1	0.07	77	>0.05
	Pre-Exercise	0.4 ± 0.0	0.4 ± 0.1	0.0	0.31	77	>0.05
	100W	1.7 ± 0.0	1.7 ± 0.5	0.0	0.27	77	>0.05
	150W	2.2 ± 0.1	2.2 ± 0.6	0.0	1.14	77	>0.05
	200W	2.7 ± 0.1	2.8 ± 0.8	-0.1	1.65	77	>0.05
	250W	3.2 ± 0.3	3.3 ± 0.9	-0.1	0.54	77	>0.05
	300W	3.6 ± 0.1	3.8 ± 1.2	-0.2	3.16	77	<0.05
	Wmax	4.2 ± 0.3	4.3 ± 1.2	-0.1	1.98	77	>0.05

Table S3: Haemoglobin, venous oxygen saturation and uncorrected pH and HCO_3^- values in control and KE conditions at rest and during incremental intensity exercise (n=12) following KE or control drinks. All data are means ± SEM. Hb = haemoglobin, SvO_2 = venous blood saturation, vpH = venous pH, $vHCO_3^-$ = venous bicarbonate

	**Measurement**	**Ketone Ester**	**Control**
**Hb (g/dL)**	Baseline	13.8 ± 0.4	14.1 ± 0.4
	Pre-Exercise	14.4 ± 0.5	14.2 ± 0.4
	100W	15.0 ± 0.4	15.1 ± 0.3
	200W	14.9 ± 0.5	15.4 ± 0.3
	300W	15.8 ± 0.3	15.9 ± 0.3
	Wmax	15.9 ± 0.3	16.2 ± 0.4
**SvO2 (%)**	Baseline	85.0 ± 5.1	79.6 ± 5.0
	Pre-Exercise	78.0 ± 5.3	82.5 ± 4.8
	100W	59.1 ± 6.6	65.2 ± 5.7
	200W	79.8 ± 4.0	83.9 ± 4.7
	300W	80.1 ± 3.9	85.3 ± 3.3
	Wmax	76.9 ± 3.7	81.4 ± 4.5
**vpH**	Baseline	7.39 ± 0.01	7.39 ± 0.01
	Pre-Exercise	7.35 ± 0.11	7.40 ± 0.01
	100W	7.30 ± 0.01	7.36 ± 0.01
	200W	7.30 ± 0.03	7.37 ± 0.01
	300W	7.28 ± 0.03	7.33 ± 0.02
	Wmax	7.20 ± 0.01	7.33 ± 0.01
**$vHCO_3^-$ (mM/L)**	Baseline	24.1 ± 0.4	24.5 ± 0.5
	Pre-Exercise	22.0 ± 0.5	24.4 ± 0.5
	100W	21.1 ± 0.4	24.3 ± 0.3
	200W	21.2 ± 0.6	23.9 ± 0.5

300W	19.1 ± 1.1	20.9 ± 1.2
Wmax	15.2 ± 0.4	16.1 ± 1.2
